# Understanding thio-effects in simple phosphoryl systems: role of solvent effects and nucleophile charge†
†Electronic supplementary information (ESI) available: A breakdown of calculated activation free energies shown in Table 1, as well as absolute energies and Cartesian coordinates of all key species in this work are presented as ESI. See DOI: 10.1039/c5ob00309a



**DOI:** 10.1039/c5ob00309a

**Published:** 2015-03-23

**Authors:** Alexandra T. P. Carvalho, AnnMarie C. O'Donoghue, David R. W. Hodgson, Shina C. L. Kamerlin

**Affiliations:** a Science for Life Laboratory , Department of Cell and Molecular Biology , Uppsala University , BMC Box 596 , SE-751 24 , Uppsala , Sweden . Email: kamerlin@icm.uu.se; b Biophysical Sciences Institute , Durham University , South Road , Durham DH1 3LE , UK; c Department of Chemistry , Durham University , South Road , Durham DH1 3LE , UK

## Abstract

Recent experimental work (*J. Org. Chem.*, 2012, **77**, 5829) demonstrated pronounced differences in measured thio-effects for the hydrolysis of (thio)phosphodichloridates by water and hydroxide nucleophiles. In the present work, we have performed detailed quantum chemical calculations of these reactions, with the aim of rationalizing the molecular bases for this discrimination. The calculations highlight the interplay between nucleophile charge and transition state solvation in S_N_2(P) mechanisms as the basis of these differences, rather than a change in mechanism.

## Abstract 2

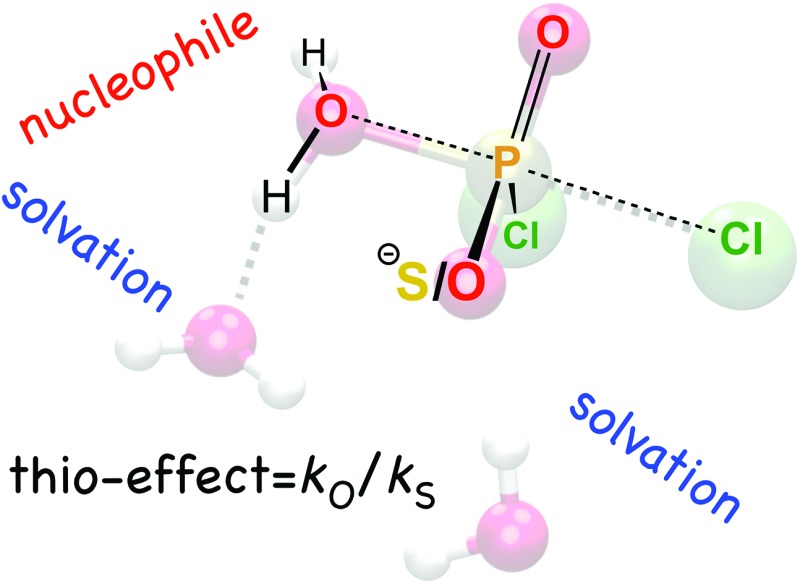
Detailed quantum chemical calculations provide insight on the origin of large differences in experimental thio-effects for the hydrolysis of (thio)phosphodichloridates by water and hydroxide nucleophiles.

## Introduction

Phosphoryl transfer reactions are crucial to biology, being involved in a range of processes from ATP synthesis to maintaining the integrity of our genetic material.^1,2^ The hydrolyses of these compounds are mechanistically complex, as they can proceed through multiple pathways ranging from fully dissociative (D_N_ + A_N_), concerted (A_N_D_N_) to fully associative processes (A_N_ + D_N_), depending on whether the reactions are driven by bond formation to the nucleophile or bond cleavage from the leaving group. As a result of this complexity, it can be challenging to unambiguously assign a reaction mechanism to a given system, and it is non-trivial to distinguish between potentially similar transition states,^2–5^ whether computationally or experimentally.

**Fig. 1:**
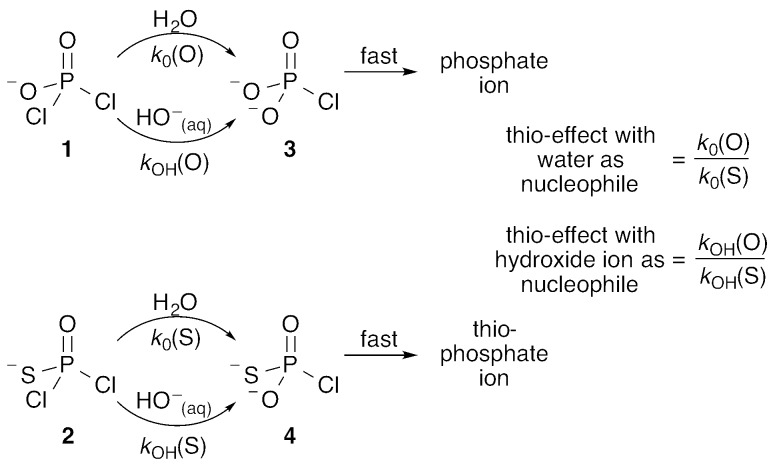
The water and hydroxide reactions of the phosphodichloridate ion **1** and thiophosphodichloridate ion **2**, and the definitions of the corresponding “thio-effects”.

**Fig. 2:**
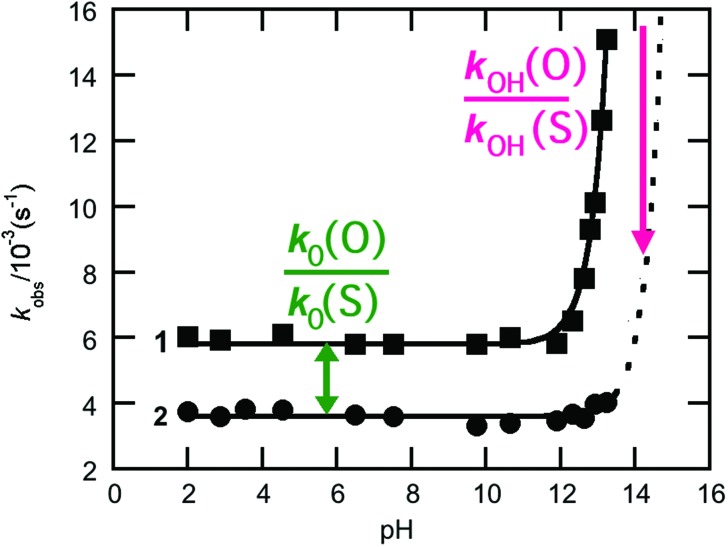
*k*_obs_-pH rate profiles for the hydrolysis of the phospho- (**1**, **■**) and thiophosphodichloridate (**2**, ****) ions considered in this work. The relevant thio-effects are defined on the pH rate profile.

One particularly valuable approach is the use of thio-substitution experiments (see ref. 1 and references cited therein), in which one of the oxygen atoms of a phosphate ester is substituted for a sulfur atom. This single atom perturbation can have quite a dramatic effect on charge distribution and bond-lengths around the phosphorus center, altering solvent interactions and the overall rate constant of reaction. In a recent study,^6^ we explored the hydrolyses of dichloridates **1** and **2** (Fig. 1), which are simple phosphoryl compounds that represent the first hydrolysis products of the industrially important bulk chemicals POCl_3_ and PSCl_3_ (Fig. 1). In the case of the phosphodichloridate ion, **1**, we observed a plateau in the reactivity up to pH ∼ 12, with *k*_0_ = (5.7 ± 0.2) × 10^–3^ s^–1^ for the pH-independent uncatalyzed hydrolysis reaction with solvent water as the nucleophile (Fig. 2). In contrast, the thiophosphodichloridate ion, **2**, showed essentially constant reactivity across the pH range from ∼2 to ∼13, with *k*_0_ = (3.6 ± 0.06) × 10^–3^ s^–1^. This translates to an observed “thio-effect” (*k(*O)/*k*(S)) of 1.6 for the water reaction. In the corresponding case of an anionic nucleophile (*i.e.* the hydroxide reactions), we observed increased reactivity above pH ∼ 12 for the phosphodichloridate ion **1** with *k*_OH_ = (5.6 ± 0.2) × 10^–2^ M^–1^ s^–1^. The thiophosphodichloridate ion **2**, in contrast, showed limited reactivity towards hydroxide ion under the conditions of our experiments, corresponding to an observed thio-effect of >31 (as a lower-limit) for the reactions of hydroxide ion with dichloridates **1** and **2**.

Previous work from York and co-workers on the role of solvation on thio-effects for a number of related phosphoryl transfer reactions,^7,8^ showed compensatory solute–solvent interactions upon moving from the gas-phase to solution for the attack of an anionic nucleophile, methoxide ion, on a cyclic (thio)phosphate, leading to similar activation barriers to oxy- and thio-reactions.^8^ To assess whether similar effects are at play for phosphodichloridate substrates **1** and **2**,^6^ we have compared water and the hydroxide anion as nucleophiles using density functional theory methods. We considered only the kinetically relevant, rate-determining steps between phosphodichloridate **1** and phosphomonochloridate **3**, and thiophosphodichloridate **2** and thiophosphomonochloridate **4** in our calculations. In the case of the water reactions, we modelled the systems as solvent-assisted reactions involving proton abstraction from the attacking nucleophile to an additional water molecule upon P–O bond formation. Note also that, as demonstrated in ref. 9, the inclusion of a minimum of at least two water molecules appears necessary to obtain computationally meaningful results for the hydrolysis of related phosphate monoester dianions, and, in the case of monoanionic phosphate diesters, two or three additional explicit water molecules were shown to be sufficient to provide reasonable agreement with experimental observables.^10^ Therefore, for both water and hydroxide ion reactions of substrates **1** and **2**, a further two explicit water molecules were symmetrically included in the calculations to allow for stabilization of nucleophile and leaving group, as well as any potentially relevant proton transfers from the nucleophile.

A major challenge when attempting to computationally reproduce the experimental thio-effects in a quantitative manner is the very small differences in free energy involved between the reactions of **1** and **2** with a given nucleophile. In our case, thio-effects of 1.6 and >31 correspond to ΔΔ*G* ^‡^ values of only 0.3 and >2 kcal mol^–1^ respectively between the water and hydroxide reactions. The current “gold-standard” for such calculations is to fall within 1 kcal mol^–1^ of experimentally observed values. As very small errors in calculated free energies can lead to very large errors in the corresponding calculated thio-effects, we are not aiming for *absolute* quantitative agreement between the calculated and experimental thio-effects. Rather, our goal is to reproduce and rationalize the *effect of the change in nucleophile from water to hydroxide* on the relative reactivities of the phosphodichloridate ion **1** and thiophosphodichloridate ion **2**. We analyse all stationary points for the first step of the mechanisms with the different nucleophiles, regarding energies, charges and bond orders. We also provide the activation strain analysis of the relevant structures.

## Methodology

All calculations in this work were performed using the dispersion corrected M06-2X density functional^11^ (chosen for its excellent performance in our recent studies of related compounds), the SMD solvation model^12^ (SMD is a universal solvent model, where the “D” denotes density, indicating that the full solute electron densities are used without the need for defining partial atomic charges^12^), and the 6-31+G* basis set for initial geometry optimizations and IRC calculations, followed by the larger 6-311++G** basis set for single point energies. The zero point corrections to the energies and entropies were obtained from the vibrational frequencies, and added to the calculated energies. Bond orders were calculated based on the Wiberg bond^13^ index using natural bond orbital analysis,^14^ and partial charges presented in this work are calculated using Merz–Kollman charges^15,16^ obtained at the same level of theory as the single point energy calculations. All calculations were performed using the Gaussian09 program.^17^

For all systems, two extra explicit water molecules were included in the simulation in addition to the reacting atoms, in order to stabilize the nucleophile and leaving group, and allow for any possible necessary proton transfers. In order to ensure that the relative positions of the water molecules to the reacting atoms are as close as possible when moving from the phosphodichloridate **1** to the thiophosphodichloridate **2**, we obtained the transition state for the hydrolysis of the thio- phosphodichloridate **2** by simply substituting O for S at the transition states, and re-optimizing.

As with our previous work,^18,19^ all calculations involving the hydroxide ion were revised by adding a standard correction of –7.2 kcal mol^–1^ to the solvation free energy of hydroxide ion, to take into account the artificial destabilization of the ground state arising from the under-solvation of the hydroxide ion (see detailed discussion in ref. 18, 19, and references cited therein). It should be emphasized that this correction provides better agreement with experimental values for solvation and activation free energies. As this is a constant correction for both phosphodichloridate **1** and thiophosphodichloridate **2** ions, it does not affect any discussion relating to differences in activation barriers.

## Results and discussion

**Fig. 3:**
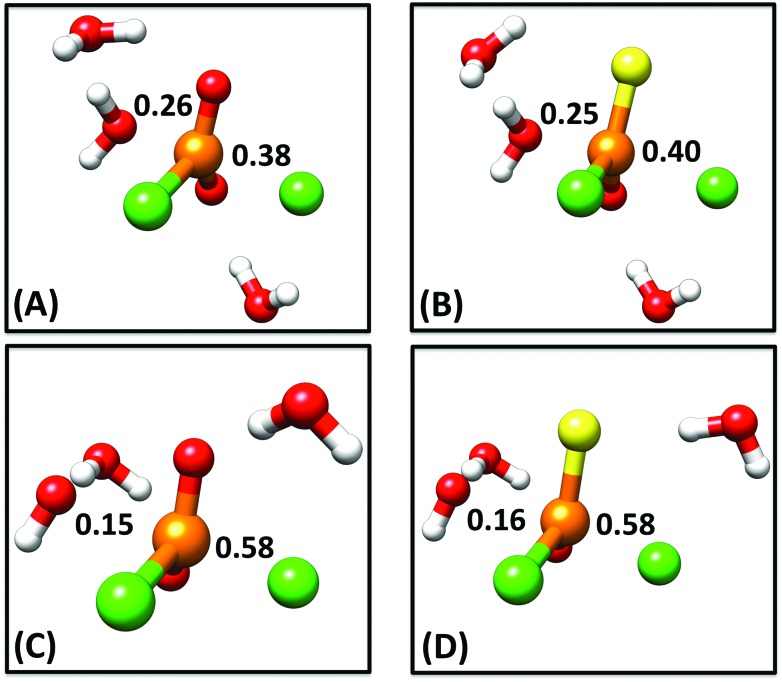
A comparison of optimized transition state geometries for (A) the water reaction of phosphodichloridate **1**, (B) the water reaction of thiophosphodichloridate **2**, (C) the hydroxide reaction of phosphodichloridate **1** and (D) the hydroxide reaction of thiophosphodichloridate **2**. Bond orders to the incoming nucleophile and departing group are labeled on all structures, and were calculated as outlined in the Methodology section. Partial bonds have been omitted from all structures for clarity. All geometry optimizations were performed at the M06-2X/6-31+G*/SMD level of theory.

The monochloridate products **3** and **4** are not observed in the hydrolyses of **1** and **2**, thus it can be assumed that the subsequent reaction of these species to inorganic (thio)phosphate is fast, and the first, rate-limiting hydrolysis step is the only necessary focus of the present work. Therefore, as our starting point, we generated 1-D free energy surfaces for the hydroxide and water reactions of compounds **1** and **2** as described in the Methodology section. This was done by first performing a rough potential energy scan along the P–O_nuc_ distance to identify an approximate transition state for each reaction, after which we performed an unconstrained optimization to obtain the true transition state (at this level of theory). We then followed the intrinsic reaction coordinate (IRC)^20^ from this transition state in both reactant and product directions, and finally performed unconstrained geometry optimizations on the endpoints of the IRC calculations to obtain the relevant reactant and product complexes (coordinates of all stationary points can be found in the ESI†). Comparisons of the optimized transition states and resulting free energy profiles for all four reactions can be found in Fig. 3. The resulting energetics and both calculated and experimental rate constants are shown in Tables 1 and S1.† Cartesian coordinates of all key stationary points are also provided as ESI.†

**Table 1:** A comparison of calculated and experimental energetics and kinetics for the water and hydroxide reactions of dichloridates **1** and **2** (Fig. 1)^*a*^

System	Δ*g* ^‡^_calc_/kcal mol^–1^	Δ*g* ^‡^_exp_/kcal mol^–1^	*k*_calc_	*k*_exp_	(*k*(O)/*k*(S))_calc_	(*k*(O)/*k*(S))_exp_
**Water reactions**						
**1**	22.5	20.5	2.6 × 10^–4^ s^–1^	5.7 × 10^–3^ s^–1^	10.5	1.6
**2**	23.9	20.8	2.9 × 10^–4^ s^–1^	3.6 × 10^–3^ s^–1^		

**Hydroxide reactions**						
**1**	16.8	19.2	3.7 M^–1^ s^–1^	5.6 × 10^–2^ M^–1^ s^–1^	47.4	>31
**2**	19.1	>21.2	7.8 × 10^–2^ M^–1^ s^–1^	<1.8 × 10^–3^ M^–1^ s^–1^		

^*a*^All energies are given in kcal mol^–1^. Δ*g* ^‡^_calc_ and Δ*g* ^‡^_exp_ denote calculated and experimental activation free energies respectively. *k*_calc_ and *k*_exp_ denote calculated and experimental rate constants. The rate constants for the water reactions (*k*_0_) are in units of s^–1^, and, for the corresponding hydroxide reactions (*k*_OH_) in M^–1^ s^–1^, and calculated rate constants and experimental activation barriers were obtained from the corresponding experimental/calculated values using transition state theory. *k*(O)/*k*(S) denotes the “thio-effect” obtained by taking the ratio between the rate constants for the hydrolyses of dichloridates **1** and **2** respectively. The difference between the activation free energies of compounds **1** and **2** are calculated to be 1 kcal mol^–1^ for the water reaction (experimental difference 0.3 kcal mol^–1^), and 2.3 kcal mol^–1^ for the hydroxide reaction (experimental difference >2 kcal mol^–1^).

From our calculations, we observe that water attack on phosphodichloridate **1** leads to a free energy barrier of 22.5 kcal mol^–1^, whereas water attack on thiophosphodichloridate **2** amounts to a free energy barrier of 23.9 kcal mol^–1^. For the hydroxide reactions, the corresponding free energy barriers are 16.8 and 19.1 kcal mol^–1^ for **1** and **2** respectively. Once again, we would like to emphasize that a major challenge in this work is that comparatively small differences in free energy, close to the 1 kcal mol^–1^ “gold-standard” of computational chemistry (which many, if not most, theoretical studies fall short of), can translate to very large errors in rate constants due to the exponential relationship between these two parameters. For example, the experimental thio-effect of 1.6 observed for the water reaction (Table 1) corresponds to an activation free-energy difference of 0.3 kcal mol^–1^ between the reactions of compounds **1** and **2**. Our calculations provide a ΔΔ*G* ^‡^ of 1.4 kcal mol^–1^ for this reaction, which is in reasonable agreement with experiment, within the limitations of current computational models. However, based on the exponential relationship between rate constants and activation free energies, this difference translates to a larger thio-effect of 10.4 instead of 1.6. Similarly, our calculated ΔΔ*G* ^‡^ of 2.4 kcal mol^–1^ for the hydroxide reactions of **1** and **2** is consistent with the experimental estimate of >2 kcal mol^–1^ that translates to a large calculated thio-effect of 47.4 compared to the experimental estimate of >31 (see Tables 1 and S1†). Despite the offset from absolute thio-effects, we are able to capture the discrimination between the two reactions extremely well, even though this is a very subtle effect in terms of relative activation free energies. Thus credence can be given to the structural and atomic level explanations our calculations yield in discerning the origins of these effects. Experimental rate constants are shown in Tables 1 and S1.† Cartesian coordinates of all key stationary points are also provided as ESI.†

**Fig. 4:**
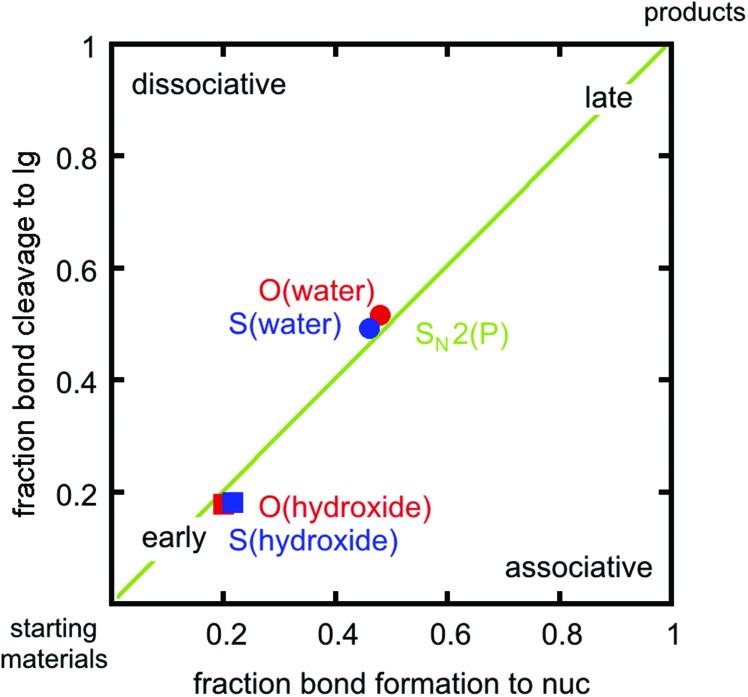
A comparison for the water and hydroxide reactions of phospho- and thiophosphodichloridates **1** and **2** on a More O'Ferrall–Jencks diagram, utilizing fractional degrees of bond formation. The nucleophile and leaving group are denoted by nuc and lg respectively.

In addition to overall energetics, we also examined bond-distances, bond-orders and partial charge distributions for key stationary points (Tables 2 and 3), and the breakdowns of the calculated activation free energies for each compound (Table S1†). From the changes in bond order/bond distances, it can be seen that for both the hydroxide and water reactions, the phospho- and thiophosphodichloridates **1** and **2** have similar geometric parameters at the relevant transition states. However, the reactions differ (Fig. 3 and Tables 2 and 3), with more synchronous transition states for the water reactions of **1** and **2**, and much earlier transition states for the hydroxide reactions, as clearly illustrated in the More O'Ferrall–Jencks diagram^21,22^ in Fig. 4, which charts fraction of bond formation/cleavage at the transition states. Additionally, geometric changes to any of the non-bridging oxygen atoms, the sulfur atom, or the spectator chlorine atom are absent. Instead, the key discernable geometric changes apply only to the incoming nucleophile, the departing chloride ion and in solute–solvent interactions at the different transition states.

**Table 2:** A comparison of calculated bond distances, bond orders and partial charges for the water reaction of dichloridates **1** and **2** (Fig. 1)^*a*^

System	Phosphodichloridate **1**			Thiophosphodichloridate **2**		
	Reactant state	Transition state	Product state	Reactant state	Transition state	Product state
**Bond distances**						
P–O_nuc_	3.60	1.97	1.64	3.69	2.01	1.65
P–Cl_lg_	2.06	2.52	5.14	2.08	2.54	4.90
P–O_nb_(**1**)	1.49	1.49	1.49	1.50	1.50	1.50
P–O_nb_(**2**)/P–S	1.50	1.50	1.49	1.95	1.96	1.95
P–Cl	2.06	2.07	2.08	2.07	2.09	2.10

**Absolute bond orders**						
P–O_nuc_	0.0044	0.2604	0.5378	0.0040	0.2464	0.5317
P–Cl_lg_	0.7866	0.3801	0.0005	0.7871	0.3997	0.0006
P–O_nb_(**1**)	1.1415	1.1926	1.2403	1.1074	1.1595	1.2155
P–O_nb_(**2**)/P–S	1.1780	1.1845	1.2211	1.3898	1.3905	1.4186
P–Cl	0.7873	0.7759	0.7700	0.7699	0.7571	0.7582

**Fractional degree of bond formation/cleavage** ^*b*^						
P–O_nuc_ (formation)	0.00	0.480	1.00	0.00	0.459	1.00
P–Cl_lg_ (cleavage)	0.00	0.517	1.00	0.00	0.493	1.00

**Partial charges**						
O_nuc_	–1.063	–0.678	–0.582	–1.001	–0.712	–0.480
P	0.732	0.944	1.097	0.128	0.344	0.318
Cl_lg_	–0.157	–0.709	–0.958	–0.086	–0.667	–0.952
O_1_	–0.628	–0.660	–0.752	–0.451	–0.527	–0.577
O_2_/S	–0.664	–0.653	–0.779	–0.340	–0.334	–0.409
Cl_sp_	–0.186	–0.172	–0.269	–0.126	–0.105	–0.178

^*a*^Bond distances are provided in Å. Bond orders were obtained from Wiberg bond indices,^13^ by performing natural bond orbital analysis.^14^ Partial charges are Merz–Kollman charges^15,16^ using the 6-311++G** basis set, the M06-2X functional and the SMD implicit solvent model. Note that the non-zero bond orders for the P–O_nuc_ bond at the reactant state are due to the fact that our reference point is a geometry optimized reactant complex rather than the fragments at infinite separation. Shown here are also calculated bond orders normalized to the reactant state.

^*b*^For the construction of a More-O'Ferrall–Jencks diagram, fractional degrees of bond formation/cleavage were calculated. Values of P–O_nuc_ = 0.00 (formation) and P–Cl_lg_ = 0.00 (cleavage) were used in reactant states, and P–O_nuc_ = 1.00 (formation) and P–Cl_lg_ = 1.00 (cleavage) were used in the product states. Transition state values were calculated by interpolation based on these values.

**Table 3:** A comparison of calculated bond distances, bond orders and partial charges for the hydroxide reaction of dichloridates **1** and **2** (Fig. 1)^*a*^

System	Phosphodichloridate **1**			Thiophosphodichloridate **2**		
	Reactant state	Transition state	Product state	Reactant state	Transition state	Product state
**Bond distances**						
P–O_nuc_	3.71	2.47	1.61	3.95	2.52	1.61
P–Cl_lg_	2.07	2.18	5.07	2.08	2.21	4.67
P–O_nb_(**1**)	1.49	1.49	1.50	1.50	1.50	1.51
P–O_nb_(**2**)/P–S	1.50	1.50	1.50	1.96	1.97	1.97
P–Cl	2.07	2.07	2.09	2.08	2.09	2.11

**Absolute bond orders**						
P–O_nuc_	0.0066	0.1464	0.7062	0.0065	0.1573	0.7024
P–Cl_lg_	0.7112	0.5838	0.0004	0.7129	0.5839	0.0006
P–O_nb_(**1**)	1.2216	1.2005	1.1349	1.1901	1.1814	1.1697
P–O_nb_(**2**)/P–S	1.1949	1.1602	1.1760	1.3694	1.3307	1.3232
P–Cl	0.7615	0.7457	0.7190	0.7778	0.7384	0.7077

**Fractional degree of bond formation/cleavage** ^*b*^						
P–O_nuc_ (formation)	0.00	0.200	1.00	0.00	0.217	1.00
P–Cl_lg_ (cleavage)	0.00	0.179	1.00	0.00	0.181	1.00

**Partial charges**						
O_nuc_	–1.307	–1.235	–0.789	–1.350	–1.242	–0.709
P	0.827	0.834	1.248	0.134	0.456	0.664
Cl_lg_	–0.191	–0.391	–0.952	–0.107	–0.367	–0.941
O_1_	–0.724	–0.706	–0.865	–0.485	–0.584	–0.698
O_2_/S	–0.648	–0.581	––0.785	–0.334	–0.347	–0.515
Cl_sp_	–0.207	–0.182	–0.315	–0.109	–0.156	–0.270

^*a*^Bond distances are provided in Å. Bond orders were obtained from Wiberg bond indices,^13^ by performing natural bond orbital analysis.^14^ Partial charges are Merz–Kollman charges^15,16^ using the 6-311++G** basis set, the M06-2X functional and the SMD implicit solvent model. Note that the non-zero bond orders for the P–O_nuc_ bond at the reactant state are due to the fact that our reference point is a geometry optimized reactant complex rather than the fragments at infinite separation.

^*b*^For the construction of a More-O'Ferrall–Jencks diagram, fractional degrees of bond formation/cleavage were calculated. Values of P–O_nuc_ = 0.00 (formation) and P–Cl_lg_ = 0.00 (cleavage) were used in reactant states, and P–O_nuc_ = 1.00 (formation) and P–Cl_lg_ = 1.00 (cleavage) were used in the product states. Transition state values were calculated by interpolation based on these values.

Thio-effects of as small as 0.1–0.3 have been observed for phosphate monoesters, in which more dissociative mechanisms dominate with good leaving groups. Increasingly larger thio-effects are observed for phosphodiesters^23–25^ (also normally expected to be S_N_2(P)) and finally these effects can be as large as 10–160 for phosphotriesters, which tend to be dominated by associative pathways (for a discussion of the provenance of these values and their interpretation, *cf.* ref. 23–27, and references cited therein). In our case, the calculations reveal two A_N_D_N_ (S_N_2(P)) mechanisms for the reactions of dichloridates **1** and **2** with water and hydroxide attack respectively. The key difference lies in the hydroxide reaction, which tends towards earlier transition states than the corresponding water reaction. In our calculations, the sum of the normalized P–O_nuc_ and P–Cl_lg_ fractional bond orders at the transition state are approximately 1.02 and 0.96 for the hydroxide and the water reactions respectively (*cf.* Tables 2 and 3, note also the differences in bond order at the product state). Interestingly, the transition states obtained for both the hydroxide reactions are very similar in nature to transition states we have previously obtained for the corresponding hydroxide attack on arylphosphate diesters,^28^ fluorophosphates^29^ and even arylsulfonate monoesters.^18^ This is consistent with the larger thio-effects that we observe for the hydroxide reactions of dichloridates **1** and **2**.

### Origin of thio-effects

What then is the origin of the thio-effects for the different nucleophiles? The answer appears to lie partly in the electrostatics of the reaction where the biggest differences between the water and hydroxide reactions are seen in the changes in charge distribution upon moving from the ground state to the transition state (Tables 2 and 3), and the associated changes in solvation requirements (Table S1†). A comparison of the changes in the partial charges on key atoms upon moving from reactants to transition states (Tables 2 and 3) reveals greater changes on the central phosphorus atoms and the spectator oxygen atoms in the case of the hydroxide reactions. In the case of the thiophosphodichloridate **2**, the phosphorus atom gains a more positive charge relative to the phosphodichloridate ion **1**. For the corresponding water reactions, there is less build-up of negative charge on the spectator oxygen atoms. Clearly, one would expect the differences in these transition states to translate also to changes in solvation patterns. This is supported by Table S1,† which shows that for the hydroxide reactions, where the largest thio-effect is observed, both transition states appear to be substantially solvent destabilized (we remind the reader that in both cases we have corrected for undersolvation of the hydroxide anion, see the Methodology section). This can be expected from a reaction involving nucleophilic attack of an anion on a monoanion, where the individual anionic species are better solvated than the dianionic transition state complex. However, the thio-substituted compound has even greater solvent destabilization by about 2.4 kcal mol^–1^, which also aligns with the observed thio-effect. In contrast, for the water reactions, both transition states are solvent stabilized, and the observed difference in calculated activation free energies is due to subtle differences between the calculated gas-phase energetics and solvation free energies. Although different in nature, these effects are near compensatory in magnitude (Table S1†), and result in similar calculated activation barriers for the water reactions of the two compounds.

### Activation strain analysis

To further analyse the discrimination between the water and hydroxide reactions, we performed activation strain analyses^30^ on all four reactions considered in this work (Table 4). The main difference between the reactivities of the phosphodichloridate **1** and thiophosphodichloridate **2** towards water as nucleophile is that the latter reaction is slightly less strained (Δ*E*_strain_), where this reduction in strain is counterbalanced by less favourable interaction energy (Δ*E*_EI_, which includes electrostatic and solvation effects) (Table 4). This compensation reduces the difference in total energies for the two water reactions. In contrast, in the hydroxide reactions, all energy contributions are less favourable for the thiophosphodichloridate **2**, leading to the observed difference in activation free energies. Therefore, the observed thio-effect appears to be a result of the interplay between electrostatics and internal strain of the reacting system. However, the magnitude of ΔΔ*E*_EI_ between the reactant complexes of **1** and **2** is larger than the ΔΔ*E*_strain_ for the water and hydroxide reactions, respectively, suggesting a larger role for electrostatic and solvation effects. In general for A_N_D_N_ mechanisms in phosphoryl systems, our results suggest that larger thio-effects, as observed for the hydroxide reactions with **1** and **2**, can be expected when changes in strain energies are not counterbalanced by electrostatic and solvation effects.

**Table 4:** Activation strain analysis of the reactant complexes and transition state structures for the water and hydroxide reactions of compounds **1** and **2** ^*a*^

System	*d*_nuc–P_	Δ*E*_strain_	Δ*E*_EI_	Δ*E*_total_
**Water reaction**				
*Phosphodichloridate* **1**				
Reactant state	3.61	0.0	0.0	
Transition state	1.99	32.5	–13.6	18.9
*Thiophosphodichloridate* **2**				
Reactant state	3.69	0.0	0.0	
Transition state	2.03	27.7	–7.0	20.7

**Hydroxide reaction**				
*Phosphodichloridate* **1**				
Reactant state	3.71	0.0	0.0	
Transition state	2.50	10.4	–3.4	7.0
*Thiophosphodichloridate* **2**				
Reactant state	3.95	0.0	0.0	
Transition state	2.55	12.2	–1.3	11.0

^*a*^ *d*_nuc–P_ denotes the distance between the oxygen of the attacking nucleophile and the phosphorus atom, Δ*E*_strain_ denotes the difference in strain contribution upon moving from the reactant to the transition state, Δ*E*_EI_ denotes the difference in interaction energy upon moving from the reactant to the transition state, and Δ*E*_total_ denotes the difference in total energy upon moving from the reactant to the transition state, but before the addition of zero point energy and entropy corrections, as well as before the inclusion of the 7.2 kcal mol^–1^ correction to the solvation free energy of the hydroxide ion (see Tables 1 and S1 and the Methodology section). All energies are provided in kcal mol^–1^ and distances in Å.

We also found lower contributions from distortion (strain) relative to the reactants in the hydroxide reactions, aligning with the earlier TSs with the hydroxide nucleophile further away from the substrate, leading to reduced levels of distortion (note from Tables 2 and 3 that while the bond *orders* involved are very similar, the bond *distances* involved are significantly different). In the case of the water reactions, the larger nucleophile and more synchronous TSs, with more cleavage to the leaving groups (see Fig. 4), appear to lead to much higher strain energies. To verify this hypothesis, we performed an additional energy decomposition calculation on a structure along the calculated IRC for the water reaction that was closer in structure to the TS of the hydroxide reaction for each of compounds **1** and **2**, with P–O_nuc_ distances of 2.48 Å in the case of the phosphodichloridate, and 2.46 Å in the case of the thiophosphodichloridate **2**. This gave a Δ*E*_strain_ of 5.2 kcal mol^–1^ for phosphodichloridate **1** and 7.7 kcal mol^–1^ for thiophosphodichloridate ion **2**, which is more similar to the values obtained at the TS of the hydroxide reaction, suggesting that the differences in the magnitude of Δ*E*_strain_ between the two reactions do indeed come from differences in transition state geometries (which are then amplified by the substitution of O for S).

## Overview and conclusions

Based on our computational work, it appears that the reactive (thio)phosphodichloridates **1** and **2** behave in an equivalent manner to phosphate diesters (see ref. 1 and references cited therein), proceeding from synchronous to slightly more asynchronous A_N_D_N_ transition states as a function of normalized bond orders to the incoming nucleophile and departing leaving group, and with high sensitivity to the nature of the nucleophile. In the present case, changes in environment and solute–solvent interactions allow for shifts in the natures of the transition states. It is primarily this interplay between the charge of the nucleophile, the solvation of transition states and the associated differences between internal strain and solvation, which give rise to the differences in the experimentally observed thio-effects.

Recent years have seen some very elegant computational studies of thio-effects for phosphoryl transfer reactions that have provided good qualitative agreement with experimental observables.^7,8,31–34^ While the very small differences in free energy involved mean that our calculated thio-effects are offset from the experimental values, we have been able to reproduce an experimentally observed energetic discrimination of only 1.7 kcal mol^–1^ (in terms of differences of ΔΔ*G* ^‡^) between the water and hydroxide reactions to well within 1.0 kcal mol^–1^ accuracy. This is in part due to the quality of the exchange–correlation functional used, which we have previously shown to provide proficient descriptions of reactions involving P- and S-centers.^9,18^ Thereafter, we combined this with our recently presented solvent assisted transition state for the analogous hydrolysis of phosphate monoesters.^9^ These factors lead us to have greater confidence in our computational observables, and our work demonstrates that theory can provide quantitative predictions using computationally cheap DFT calculations (compared to higher level QM or QM/MM calculations) and simple, implicit solvation.

Interestingly, we do not observe the compensatory behaviour between gas-phase activation free energies and solvation effects observed by York and co-workers^7,8^ for the hydroxide reactions, which would be closer in nature to their systems that involve reaction of anionic methoxide, albeit for different phosphoryl substrates. Rather, for compounds **1** and **2**, we observe a similar compensatory effect between gas-phase and solution energetics only for the reactions of the neutral water nucleophile, leading to a thio-effect that is substantially smaller than that calculated for the hydroxide reaction, in qualitative agreement with the experimental data for these systems. In contrast to the present study, however, the cyclic (thio)phosphate system studied by York and co-workers reacts by an addition-elimination (A_N_ + D_N_) mechanism, proceeding *via* a 5-membered phosphorane intermediate. This contrasting observation suggests that the response to nucleophile charge is also intrinsically dependent on substrate and mechanism.

Clearly, our present work, in combination with the recent experimental data^6^ and previous theoretical studies on related systems,^7,8^ make a strong case for the role of solvent interactions and nucleophile charge in determining observed thio-effects for a given substrate and mechanism. Our results also highlight that their qualitative interpretation is far from trivial. We believe, nevertheless, that computational work can provide fundamental insight into the molecular interpretation of these effects in the characterization of mechanisms, and we demonstrate herein the ability of our calculations to describe the underpinning molecular bases for the origins of the observed thio-effects. Our approach thus provides a valuable mechanistic tool for the interpretation of thio-effect data.
