# Supplementary material for: Understanding thio-effects in simple phosphoryl systems: role of solvent effects and nucleophile charge
Source: Org Biomol Chem. 2015 Mar 23;13(19):5391–8. doi: 10.1039/c5ob00309a (PMC4425225; doi:10.1039/c5ob00309a)
Supplement: Supplementary file 1 [file OB-013-C5OB00309A-s001.pdf]

Electronic Supplementary Material (ESI) for Organic & Biomolecular Chemistry.
This journal is © The Royal Society of Chemistry 2015

Supporting Information for:

Understanding Thio-Effects in Simple Phosphoryl Systems: Role of Solvent Assistance and Nucleophile Charge

Alexandra T. P. Carvalho, AnnMarie C. O'Donoghue[2,3] , David R. W. Hodgson[2,3] and Shina C. L. Kamerlin*,[1,2]

1. Science for Life Laboratory, Department of Cell and Molecular Biology, Uppsala University, BMC Box 596, SE-751 24, Uppsala, Sweden. 2. Biophysical Sciences Institute, Durham University, South Road, Durham DH1 3LE, UK. 3. Department of Chemistry, Durham University, South Road, Durham DH1 3LE, UK.

Corresponding author email address:

kamerlin@icm.uu.se

Table of Contents

Energy Breakdown for the Systems Examined in this Work………….…………..…..….S2

Absolute Electronic Energies and Entropies for the Hydrolyses of Phospho- and Thiophosphodicloridate Ions **1** and **2**……………………………………………………..S3

Cartesian coordinates of key stationary points……………………………………………S4

Table S1: Breakdown of Different Contributions to the Total Calculated Free Energies for the Different Reactions Examined in This Work[a]

System	ΔE_{gas}	$\Delta\Delta G_{solv}$	ΔZPE	$-T\Delta S$	ΔG_{calc}	ΔG_{exp}
**Water Reaction**						
Phosphodichloridate						
**Reactant State**	0.0	0.0	0.0	0.0	0.0	
**Transition State**	32.7	-13.8	0.6	3.0	22.5	20.5
**Product State**	80.0	-77.0	1.7	1.0	5.7	
Thiophosphodichloridate						
**Reactant State**	0.0	0.0	0.0	0.0	0.0	
**Transition State**	35.4	-14.7	0.5	2.7	23.9	20.8
**Product State**	79.4	-76.2	1.3	1.0	5.5	
**Hydroxide Reaction**						
Phosphodichloridate						
**Reactant State**	0.0	0.0	0.0	0.0	0.0	
**Transition State**	-3.6	17.9	0.1	2.4	16.8	19.2
**Product State**	-60.5	23.8	2.3	0.5	-34.0	
Thiophosphodichloridate						
**Reactant State**	0.0	0.0	0.0	0.0	0.0	
**Transition State**	-2.1	20.3	-0.7	1.6	19.1	>21.2
**Product State**	-54.1	19.5	2.3	1.1	-31.2	

[a] All energies are given in kcal/mol, normalized relative to the reactant complex. ΔE_{gas} denotes the gas-phase energy of the system obtained by performing single point calculations in vacuum, $\Delta\Delta G_{solv}$ denotes the contribution from including implicit solvation, ΔZPE and $-T\Delta S$ denotes the differences in zero-point energies and entropies calculated from the vibrational frequencies, and ΔG_{calc} and ΔG_{exp} denote calculated and experimental activation and reaction free energies respectively.

Table S2: Absolute energies for all key stationary points involved in the hydrolysis of phosphodichloridates 1 and 2 by water and hydroxide nucleophiles.[a]

System	E_{el} (a.u.)	E_{ZPE} (kcal/mol)	S (kcal/mol/K^{-1})
**Water Reaction**			
Phosphodichloridate			
**Reactant State**	-1641.2903666	44.58200	118.732
**Transition State**	-1641.2791418	44.43013	111.669
**Product State[b]**	-1641.3604718	46.93260	116.812
Thiophosphodichloridate			
**Reactant State**	-1964.2385996	43.24047	121.140
**Transition State**	-1964.2211342	42.85152	115.626
**Product State**	-1964.3053846	45.60506	117.578
**Hydroxide Reaction**			
Phosphodichloridate			
**Reactant State**	-1641.7841620	53.50848	120.785
**Transition State**	-1641.7539374	54.08691	110.657
**Product State**	-1641.7798808	55.21652	117.340
Thiophosphodichloridate			
**Reactant State**	-1964.7320745	52.38140	123.104
**Transition State**	-1964.6991038	52.91553	113.955
**Product State**	-1964.7269412	53.70855	119.771

[a] Shown here are the absolute atomic energy (E_{el}, in atomic unites), zero-point energy contribution (E_{ZPE} in kcal/mol) and entropies (S, in cal/mol/K^{-1}) for all relevant stationary points.

Cartesian Coordinates of Key Stationary Points

Water Reaction

Phosphodichloridate

Reactant State

P	-0.229603	-0.510021	-0.273870
O	-0.171703	0.811871	-0.978063
Cl	-0.092342	-0.134164	1.748625
Cl	-2.155840	-1.211398	-0.427523
O	0.707334	-1.629172	-0.593730
O	2.432650	1.917049	-0.405852
H	1.505389	1.740972	-0.651804
H	2.822306	1.021451	-0.310222
H	3.562733	-0.901826	0.759541
O	3.366513	-0.749634	-0.178401
H	2.497714	-1.177290	-0.326171
H	-2.754285	2.329363	0.048516
O	-1.920504	2.818448	-0.029944
H	-1.285044	2.163706	-0.382618

Transition State

P	0.160178	-0.029551	0.440039
O	-0.746460	-0.974018	1.161450
Cl	0.246934	-0.369961	-1.598337
Cl	-1.758755	1.529002	-0.026807
O	1.001017	1.111978	0.907757
O	1.690066	-1.249267	0.649075
H	1.611860	-2.126216	0.228442
H	2.532052	-0.806918	0.321075
H	3.703006	0.438697	-1.035945
O	3.620271	0.390197	-0.069177
H	3.009355	1.113974	0.167623
H	-3.314607	-0.359589	-0.259807
O	-3.291524	-1.298977	-0.005385
H	-2.430339	-1.359675	0.455732

Product State

P	0.880249	0.331862	-0.461395
O	-0.068746	1.483141	-0.577728
Cl	0.327005	-0.734454	1.233104
Cl	-4.114497	-0.868363	-0.348870
O	1.133782	-0.654289	-1.551014
O	2.356842	0.889468	-0.009854
H	2.349201	1.639107	0.616854
H	3.535537	-0.057552	0.082730
H	4.313842	-1.191185	0.966583
O	4.316299	-0.735370	0.097579
H	4.142618	-1.417887	-0.586475
H	-3.038531	0.849921	0.446243
O	-2.543267	1.624574	0.794014
H	-1.678311	1.587367	0.339020

Thiophosphodichloridate

Reactant State

P	-0.596390	-0.296260	-0.226839
O	-0.043907	0.815739	-1.077636
Cl	-0.062219	0.167344	1.731209
Cl	-2.640748	0.022104	-0.108540
S	-0.209584	-2.168164	-0.620776
O	2.769726	1.153982	-0.679532
H	1.825585	1.133331	-0.927015
H	2.938262	0.252449	-0.332597
H	2.991444	-1.427636	1.242819
O	3.036440	-1.501179	0.275634
H	2.157435	-1.835041	0.014903
H	1.073683	3.541174	0.030541
O	0.142863	3.491912	-0.237088
H	0.022244	2.566004	-0.530043

Transition State

P	-0.028702	0.140672	-0.403378
O	0.868299	-0.435504	-1.460364
Cl	-0.146113	-1.074568	1.291651
Cl	1.983886	1.199827	0.722644
S	-1.095928	1.783414	-0.378665
O	-1.554531	-0.942054	-1.128966
H	-1.331315	-1.888276	-1.229832
H	-2.386832	-0.884431	-0.562499
H	-3.503448	-1.022111	1.280183
O	-3.654370	-0.578309	0.428613
H	-3.586203	0.371045	0.629878
H	3.357472	-0.746555	0.140387
O	3.303237	-1.464401	-0.515346
H	2.472484	-1.241631	-0.983321

Product State

P	-0.734456	-0.172678	-0.519355
O	0.106421	-1.098066	-1.353929
Cl	-0.346010	-0.700194	1.473660
Cl	3.942182	0.902942	0.479445
S	-0.712125	1.764095	-0.723476
O	-2.304141	-0.682852	-0.626525
H	-2.390386	-1.652186	-0.737996
H	-3.445907	-0.098978	0.199998
H	-4.074996	0.151929	1.689959
O	-4.223666	0.319858	0.734300
H	-4.187827	1.292796	0.606576
H	3.106620	-0.972020	-0.236039
O	2.727276	-1.816424	-0.567081
H	1.821283	-1.583739	-0.853453

Hydroxide Reaction

Phosphodichloridate

Reactant State

P	0.177679	-0.480142	-0.075243
Cl	2.047199	-1.350195	-0.225516
O	-0.830776	-1.575692	0.018472
O	0.108501	0.607906	-1.098414
Cl	0.324471	0.416960	1.779881
O	-3.010484	1.336234	0.475990
H	-2.586052	0.920696	1.241695
O	-3.277158	-0.561821	-1.218432
H	-3.174499	0.207950	-0.530580
H	-2.505383	-1.124141	-1.032787
H	2.949495	2.076116	-1.109358
O	2.124474	2.578604	-1.025611
H	1.416399	1.904645	-1.080574

Transition State

P	0.129755	0.106783	-0.236097
Cl	-1.751848	0.168680	-1.340514
O	1.095455	0.353538	-1.349690
O	-0.099033	-1.151467	0.548583
Cl	-0.290542	1.825037	0.844859
O	2.143991	0.177630	1.187512
H	2.523933	1.054148	1.023577
O	3.255006	-1.446545	-0.424028
H	2.827078	-0.770991	0.240406
H	2.744844	-1.300230	-1.235979
H	-3.237872	-1.509733	0.602882
O	-2.684811	-1.852265	1.321974
H	-1.768536	-1.615252	1.061897

Product State

P	-0.986583	0.319017	-0.352868
Cl	3.853119	-1.167862	-0.131195
O	-1.100792	-1.091630	-0.859138
O	-0.087593	1.328716	-1.005162
Cl	-0.333942	0.172492	1.631729
O	-2.440405	0.973090	-0.164068
H	-3.129515	0.263695	-0.052096
O	-3.823918	-1.354130	-0.056665
H	-4.484057	-1.414901	-0.765816
H	-2.999800	-1.712481	-0.441819
H	2.966603	0.817787	-0.183670
O	2.574838	1.719078	-0.202791
H	1.642441	1.580942	-0.470088

Thiophosphodichloridate

Reactant State

P	0.145967	-0.587930	-0.221967
Cl	2.030210	-1.472231	-0.216804
O	-0.849592	-1.696344	-0.381888
S	0.139040	0.973865	-1.402222
Cl	0.060668	0.009603	1.768624
O	-2.997907	1.763960	0.204304
H	-2.091494	1.813912	-0.138047
O	-3.578425	-0.720399	-0.137430
H	-3.335938	0.275090	-0.003117
H	-2.720584	-1.169754	-0.243868
H	3.438115	1.645383	0.233024
O	2.767192	2.344145	0.287226
H	2.020695	2.006242	-0.241569

Transition State

P	-0.198651	0.219382	0.188336
Cl	1.744554	0.823394	1.038825
O	-1.003003	0.195026	1.453650
S	0.321191	-1.319989	-0.932796
Cl	-0.405503	2.036958	-0.824155
O	-2.474131	-0.067569	-0.865838
H	-2.927744	0.731760	-0.555078
O	-3.040488	-1.854113	0.832205
H	-2.814533	-1.118026	0.121738
H	-2.416750	-1.651020	1.546787
H	3.868928	-0.975076	0.256433
O	3.607932	-1.568274	-0.465153
H	2.644436	-1.425081	-0.558472

Product State

P	-0.916204	0.371619	-0.020681
Cl	3.474632	-1.179188	0.300395
O	-0.200068	-0.610765	-0.913107
S	-0.286763	2.227800	0.139734
Cl	-0.918347	-0.527195	1.890236
O	-2.498449	0.362996	-0.329460
H	-2.768488	-0.529709	-0.684823
O	-2.577564	-2.140164	-1.343455
H	-2.962208	-2.158842	-2.234514
H	-1.619943	-1.998290	-1.479645
H	2.777469	0.439100	-0.951053
O	2.497957	1.203425	-1.505201
H	1.672592	1.513234	-1.086440
